# Physical Exercise and Executive Function in the Pediatric Overweight and Obesity Population: A Systematic Review Protocol

**DOI:** 10.3390/sports12070180

**Published:** 2024-06-26

**Authors:** Enrique Cerda-Vega, Nuria Pérez-Romero, Sergio Araya Sierralta, Antonio Hernández-Mendo, Rafael E. Reigal, Rodrigo Ramirez-Campillo, Cristian Martínez-Salazar, Rodrigo Campos-Jara, Cristián Arellano-Roco, Christian Campos-Jara, Victoria Hernández-Cifuentes, Falonn Contreras-Osorio

**Affiliations:** 1 Exercise and Rehabilitation Sciences Institute, Faculty of Rehabilitation Sciences, Universidad Andres Bello, Santiago 7591538, Chile; enrique.cerda@unab.cl (E.C.-V.); christian.campos@unab.cl (C.C.-J.); 2 Exercise and Rehabilitation Sciences Institute, Postgraduate, Faculty of Rehabilitation Sciences, Universidad Andres Bello, Santiago 7591538, Chile; nuria.perez@unab.cl; 3 Departamento de Educación Física, Universidad de Atacama, Copiapó 1531772, Chile; sergio.araya@uda.cl; 4 Department of Social Psychology, Social Anthropology, Social Work and Social Services, Universidad de Málaga, 29071 Málaga, Spain; mendo@uma.es (A.H.-M.); rafareigal@uma.es (R.E.R.); 5 Exercise and Rehabilitation Sciences Institute, School of Physical Therapy, Faculty of Rehabilitation Sciences, Universidad Andres Bello, Santiago 7591538, Chile; rodrigo.ramirez@unab.cl (R.R.-C.); victoria.hernandezcif@gmail.com (V.H.-C.); 6 Department of Physical Education, Sports, and Recreation, Pedagogy in Physical Education, School of Education and Social Sciences and Humanities, Universidad de La Frontera, Temuco 4811230, Chile; cristian.martinez.s@ufrontera.cl; 7 Hospital Mauricio Heyermann, Servicio de Psiquiatría, Angol 4650207, Chile; drodrigo.campos@gmail.com; 8 Laboratorio de Neuromecanica Aplicada, Escuela de Kinesiología, Facultad de Ciencias Médicas, Universidad de Santiago de Chile, Santiago 9170022, Chile; crisbua111@gmail.com

**Keywords:** cognition, inhibition, working memory, cognitive flexibility, mental processes, exercise, physical activity, nutrition disorders, obesity, overweight

## Abstract

Background: Executive function is often altered in overweight/obese children and adolescents, which has a negative impact on their learning and daily life. Furthermore, research has shown the benefits of physical exercise in improving cognitive performance. This protocol aims to define in a detailed and structured manner the procedures that will be conducted for the development of a systematic review of the literature aimed at evaluating the effects of physical exercise on the executive functions of children and adolescents (≤18 years) with overweight/obesity in comparison with peers in control groups. Methods: The Web of Science, PubMed, Scopus, and EBSCO databases will be searched for longitudinal studies that have at least one experimental and one control group using pre- and post-intervention measures of executive function, including working memory, inhibition, and cognitive flexibility in the pediatric population who are overweight or obese. The risk of bias and certainty of evidence will be assessed using Cochrane RoB2 and GRADE, respectively. Furthermore, Der Simonian–Laird’s random effects model will be employed for meta-analyses. The effect sizes will be calculated with 95% confidence intervals, and *p* values < 0.05 indicate statistical significance for each dimension of executive function in the different groups before and after the intervention. Discussion: The results of this review may be useful for education and health professionals to design treatment plans for overweight/obese children and adolescents, offering potential benefits related to the learning and cognitive abilities of this population. PROSPERO registration number: CRD42023391420.

## 1. Introduction

Excess weight and obesity represent significant health challenges worldwide across all age groups [1]. The World Health Organization reported that, as of 2016, over 340 million children and adolescents were either overweight or obese [2]. A 2017 study by the global Non-Communicable Disease Risk Factor Collaboration, which analyzed data from 31.5 million children and adolescents aged 5–19 years, revealed that the global obesity rate among girls rose from 0.7% in 1975 to 5.6% in 2016, and among boys, it increased from 0.9% in 1975 to 7.8% in 2016. This translates to 50 million girls and 74 million boys being classified as obese in 2016 [3]. According to the WHO, overweight and obesity are defined as “the abnormal or excessive accumulation of fat that can adversely affect health [2]”. Body mass index (BMI) is commonly used to classify children and adolescents (5–19 years of age), where one standard deviation above the median weight for age and sex is considered as being overweight, and two standard deviations above the median are considered as being obese [2]. In childhood and adolescence, obesity is associated with an increased risk of premature death [4,5], elevated blood pressure associated with cardiovascular disease and stroke [6], insulin resistance [7], type 2 diabetes mellitus [8], non-alcoholic fatty liver disease [9], and chronic low-grade inflammation [10]. In addition to its impact on physical health, obesity affects the cognitive health of children and adolescents and is associated with poorer performance in executive functions [11,12]. Weaker executive function has also been suggested as a risk factor for childhood obesity, making it relevant for prevention and treatment [13,14].

Cognition refers to the mental processes by which knowledge is acquired, processed, stored, and used [15]. Executive functioning is a cognitive ability set that allows us to plan, organize, and manipulate information, make decisions, inhibit inappropriate responses, and maintain cognitive flexibility [16]. These skills are fundamental for academic and social success, and their development is especially critical during childhood and adolescence [17]. At this stage of life, the brain is in full development and is highly sensitive to increases in body weight. This affects abilities such as reasoning, working memory, and inhibitory control [18,19,20]. Despite evidence that overweight/obese children and adolescents are found to have deficits in executive function compared to their normal-weight peers [11], there is still no consensus as to which of these skills are more impaired [21]. The mechanisms by which obesity and excess weight would affect executive function include (but are not limited to) chronic inflammation associated with excess adipose tissue [22,23,24], insulin resistance in the brain [25,26], and an altered gut microbiome [27].

In turn, physical exercise has proven to be an effective tool in the treatment of overweight/obese children and adolescents, thus favoring their cognitive development in terms of executive functions [28,29]. In this regard, Crova et al. [30] showed that overweight children may have greater inhibitory benefits than normal-weight children after a 6-month intervention following a cognitive school-based physical activity program. Consistent with these findings, Krafft et al. [31] showed improvements in executive function in overweight children after an 8-month aerobic exercise program compared to a sedentary control group. This was associated with improvements in white matter integrity as assessed through diffusion tensor imaging. The mechanisms that would explain the benefits of exercise on executive function are not well understood but may involve increased cerebral blood flow [32,33], increased production of brain-derived neurotrophic factor [34,35,36], control of cerebral glucose metabolism [37], and reduced neuroinflammation [24].

Previous systematic reviews have examined the effects of chronic physical activity interventions on cognition in overweight/obese children and adolescents [38,39,40]. Bustamante et al. [38] performed a systematic review that analyzed the effects of physical activity interventions on cognitive and academic outcomes in children (without specifying the age range) with overweight and obesity, focusing on minority groups. The review by Bustamante et al. [38] suggests that regular exercise interventions may have beneficial effects on executive function in overweight/obese adolescents. However, they found only three randomized clinical trials describing improvements, two of which specified the type of improvement, one in attention and one in planning. This highlighted the shortage of studies with good methodological quality and the need to establish efficacy and determine the optimal dose and type of exercise for these outcomes.

Martin et al. [40] conducted a systematic review of the effects of various interventions (including physical activity) on cognition and academic performance in overweight/obese children and adolescents. Based on a single study conducted in children aged 8–11 years, improvement was detected in the composite score of executive function in those who engaged in after-school physical activity compared to those who only engaged in school-based physical activity. A recent systematic review and meta-analysis [39] described improvements in the executive functions of cognitive flexibility (Standard error of the mean, SMD = 0.438; *p* = 0.088) and inhibitory control (SMD = 0.254; *p* < 0.001). However, the review [39] does not describe results on working memory (one of the main dimensions of central executive functions) or the characteristics of physical activity programs (doses). The searches were carried out until October 2019 and included only one database. This differs from the usual recommendations for the number of databases that should be used to conduct a systematic review described in the literature, which considers at least three [41,42].

Therefore, this study protocol aims to define in a detailed and structured manner the procedures that will be conducted for the development of a systematic review of the literature aimed at evaluating the effects of physical exercise and its characteristics (e.g., intensity, duration) on central executive functions in overweight/obese children and adolescents compared to their peers in active or passive control groups from longitudinal randomized and non-randomized studies.

## 2. Materials and Methods

This protocol was developed following the guidelines established by Preferred Reporting Items for Systematic Reviews and Meta-Analysis Protocols (see Supplementary Materials, Table S1) [43]. It was registered in the International Prospective Register of Systematic Reviews (CRD42023391420).

### 2.1. Eligibility Criteria

Table 1 describes the inclusion and exclusion criteria using the Patient, Intervention, Comparison, Outcome, and Study design (PICOS) strategy. In order to define a chronic exercise program, the minimum period of time described in the literature for improving executive function in obese adolescents was used, which is 4 weeks [44].

### 2.2. Search Strategy

The search will be conducted in the following databases: PubMed, Web of Science, EBSCO, and Scopus. Studies identified in bibliographic references of selected studies and related reviews identified during the preliminary search will be incorporated to meet the inclusion criteria. The search for articles in the databases will be initiated by two authors (F.C.-O. and E.C-V.) after the publication of the protocol. No filters, such as sex or date of publication, will be applied, nor will articles be excluded based on language. The search methodologies described above will be employed, incorporating medical subject headings and unstructured text expressions (see Supplementary Materials, Table S2).

### 2.3. Selection Process

The collection of studies for the review will be documented using a diagram in accordance with the PRISMA 2020 guidelines [45]. This diagram will outline the selection process and justification for exclusions. After identifying the documents in the databases, duplicates will be eliminated using bibliographic reference management software. If necessary, manual deletions supervised by one of the authors will be considered (E.C.-V.). Subsequently, two authors (F.C.-O. and E.C.-V.) will evaluate the suitability of the titles and abstracts. In addition, potentially relevant studies will be identified by examining the reference lists of included articles and reviews identified in the search. Any disparity in the decisions made by the authors will be resolved by consensus with a third author (C.C.-J.).

### 2.4. Data Extraction and Management

For each study included in the analysis, the following information will be considered: year of publication, authors, sample size, participant characteristics (including sex, age, socio-economic status, physical status at the beginning of the intervention, obesity or excess weight diagnosis, BMI, coexisting health conditions, and other treatments), characteristics of the exercise intervention (type of exercise, weekly frequency, total duration in weeks, duration of sessions in minutes, and exercise intensity expressed, for example, through metrics such as heart rate or Borg scale), level of adherence to the intervention (e.g., percentage of completed sessions relative to total or average number of sessions completed), and a description of the control condition. The dimensions of executive function assessed (i.e., working memory, inhibition, or cognitive flexibility) and the specific tasks or tests used, such as Trail Making Test-Part B [46] for cognitive flexibility, N-Back [47] for working memory, or Stroop [48] for inhibition, will be recorded.

For each outcome, the mean and standard deviation values before and after the intervention will be documented. One author (F.C.-O.) will perform the data extraction, and another (E.C.-V.) will cross-check the data, independently entering the information into separate Microsoft Excel documents. Any disagreement will be resolved by consulting a third author (R.R.-C.). In cases where the selected article does not present the data comprehensively, the communication methods established in previous literature [49,50] will be used to contact the authors. This correspondence involves a maximum of two attempts within 2 weeks. In case of no response or incomplete data, the study will be excluded from the analysis. When data are represented graphically, validated software (with a strong correlation, r = 0.99, *p* < 0.001), [51] such as WebPlotDigitizer (version 4.5, Pacifica, CA, USA; https://apps.automeris.io/wpd/ accessed on 16 May 2024), will be used to extract the numerical data needed to calculate Cronbach’s alpha.

### 2.5. Risk of Bias in Individual Studies

Similar to previous research [52,53,54], the assessment of potential bias will be performed using the second iteration of the Cochrane risk-of-bias tool (RoB2) [55]. Based on an analysis of five key aspects: randomization process, adherence to planned interventions, handling of missing outcome data, measurement of outcomes, and selection of reported outcomes, the assessment classifies trials into three risk levels: minimal risk, some concern, or high risk.

Bias assessment will be performed independently by two authors (F.C.-O. and C.C.-J.), and discrepancies will be resolved by consultation with a third author (E.C.-V.). All studies meeting the inclusion criteria will be included in the analyses, regardless of RoB2 results. However, the interpretation and discussion of the results will consider the RoB2 results.

In addition, the Grading of Recommendations, Assessment, Development, and Evaluation (GRADE) method will be applied to consolidate and assess the certainty of the evidence by categorizing it as high, moderate, low, or very low [56,57].

### 2.6. Meta-Analysis

After data extraction, the decision about whether a meta-analysis can be performed for a specific outcome will be made. Results will be analyzed for executive functions, including individual analyses for working memory, inhibition, and cognitive flexibility. For each outcome measurement, a minimum of three studies will be necessary to perform a meta-analysis [58,59], considering the typically limited number of study participants within this domain [60].

The Hedges’ g effect size (ES) will be estimated with 95% confidence and prediction intervals will be calculated from the mean and standard deviation values before and after the intervention in the experimental groups compared to the controls. When studies will provide data in formats other than mean and standard deviation, appropriate statistical conversions will be used before meta-analysis. The Der Simonian–Laird methods will be employed for the meta-analysis.

The estimated ESs will be classified as follows: <0.2 as trivial, 0.2–0.6 as small, >0.6–1.2 as moderate, >1.2–2.0 as large, >2.0–4.0 as very large, and >4.0 as extremely large [61]. The identification of non-normative ESs (e.g., >3.0) will be sought. These values may significantly affect the validity, robustness, and interpretations of the meta-analysis [62].

For studies with multiple interventions and a single control group, control group sample sizes will be divided proportionally to allow for group comparisons. The degree of heterogeneity will be assessed using the I^2^ statistic, with values indicating low (<25%), moderate (25–75%), or high (>75%) levels of heterogeneity [63].

Publication bias for continuous variables (with at least 10 studies per outcome) will be assessed using Egger’s test [55,64]. In order to account for publication bias, a sensitivity analysis will be performed using the trim-and-fill method [65] with L0 as the default estimator for missing studies [66]. A multivariate meta-regression with Der Simonian–Laird random effects model will be used to explore whether continuous moderators (such as training frequency, duration, and total number of intervention sessions) explain the effects of the intervention on the dependent variables. Meta-regression calculations require a minimum of 10 studies per covariate [45]. In addition, sensitivity analyses will be performed to validate the robustness of the summary estimates (e.g., *p*-value, ES, and I^2^).

To assess the influence of individual study results on the overall findings, automated exclusion analyses will be performed. All analyses will be performed with the comprehensive meta-analysis software (version 2, Biostat, Englewood, NJ, USA), with statistical significance set at *p* ≤ 0.05.

### 2.7. Moderators

As training characteristics may influence the main dimensions of executive function [67,68], i.e., working memory, cognitive flexibility, and inhibition, and in line with the recommendations of the Singh et al. [69] and Wassenaar et al. [70] studies, physical activity characteristics will be used as moderators, namely the intensity of the intervention program, total duration in weeks, frequency in number of weekly sessions, the duration in minutes of weekly training as well as the type of training (e.g., aerobic, resistance, high-intensity interval training, or multicomponent), level of physical activity at the beginning of the intervention, socio-economic status, and sex. Therefore, we will consider the diagnosis associated with malnutrition (obesity or being overweight) and the characteristics of the tasks performed [39].

To perform the analysis through a moderator, the median splitting technique will be used. Thus, if at least three studies provide data for a given moderator, the median will be calculated for each category. If a study includes two experimental groups with the same information for a given moderator, only one will be considered to avoid undue influence on the median calculation.

## 3. Discussion

This systematic review protocol aims to define in a detailed and structured manner the procedure that will be carried out for the development of a review of the literature that will evaluate the effects of physical exercise on the executive functions of children and adolescents with overweight/obesity in comparison with their peers in control groups. To this end, the scientific literature that describes the use of chronic exercise programs and their effects on the so-called central executive functions, that is, cognitive flexibility, working memory, and inhibition, will be reviewed in depth.

Because this type of malnutrition is a major public health problem [3] that affects physical and mental dimensions throughout life [71], understanding the effects of physical exercise on cognitive function in childhood and adolescence under conditions of being overweight/obese is of significant importance to health professionals and educators. This investigation aims to provide background information to support physical activity interventions and promote exercise as a useful tool for the management of malnutrition due to excess, thus ensuring the optimal development of children and adolescents.

To date, we found only three previous systematic reviews [38,39,40] that addressed the effects of physical activity on cognitive performance in children and adolescents with obesity or overweight, and there are still aspects that can be addressed to complement this analysis, which we have included in the current investigation. As part of these considerations, we plan to expand our search to four databases, extending the age range of participants up to 18 years and considering outcome measures for the three main dimensions of executive functions. We will also include both randomized and non-randomized controlled studies that provide pre- and post-intervention results with a control group as a means to obtain a comprehensive view of the current evidence in the literature.

In addition, moderator analysis is expected to provide greater clarity regarding the effects of physical exercise on the executive functions of children and adolescents with excess malnutrition, focusing on the characteristics of the intervention programs (i.e., the type of physical exercise, intensity of the intervention program, the total duration in weeks, frequency in number of weekly sessions, and duration in minutes of weekly training) and relevant characteristics of participants (e.g., their sex or socio-economic status). The analysis of the methodological quality and limitations of the selected studies will provide useful information for future researchers wishing to delve deeper into this topic. It will also highlight challenges that could contribute to the definition of future lines of research. The goal is to publish this systematic review in a peer-reviewed journal and to disseminate the final results to the broadest possible audience, emphasizing education and health professionals working with overweight/obese children and adolescents. The results of this review may be useful for designing treatment plans aimed at providing potential benefits related to the learning and cognitive abilities of this population.

## Figures and Tables

**Table 1:** Eligibility criteria.

	Inclusion	Exclusion
Population	Children or adolescents (≤18 years) who are overweight or obese, as determined by age- and sex-specific body mass index (BMI) percentiles, BMI standard deviation values, or waist circumference percentiles relative to a national or international reference population.	Children and adolescents with normal weight.
Intervention	Chronic exercise program, with multiple exercise sessions, at least 4 weeks long. Any type of exercise (aerobic, strength, high-intensity interval training, etc.).	Acute exercise interventions (or single session). Chronic exercise interventions that are part of a multicomponent weight loss program (e.g., diet plus exercise or bariatric surgery plus diet plus exercise).
Comparator	Passive (e.g., waiting list) or active (e.g., relaxation techniques) control condition.	Absence of control group.
Outcomes	Behavioral measures of executive function derived from composite score batteries or independent tasks designed to assess specific dimensions (e.g., working memory, inhibitory control, or cognitive flexibility).	Cognitive measures pertaining to other cognitive skills (e.g., attention, long-term memory, or visuospatial skills).
Study design	Longitudinal studies of the randomized controlled trial (RCTs), non-RCT, and controlled pre–post study types.	Case studies, cross-sectional studies, and reviews.

## Data Availability

No new data were created or analyzed in this study. Data sharing is not applicable to this article.

## Supplementary Materials

The following supporting information can be downloaded at https://www.mdpi.com/article/10.3390/sports12070180/s1, Table S1: Checklist PRISMA-P 2015; Table S2: Terms to be used in the search strategy according to each database. Ref. [43] are cited in the Supplementary Materials.

## References

[1] Williams E.P., Mesidor M., Winters K., Dubbert P.M., Wyatt S.B. (2015). Overweight and Obesity: Prevalence, Consequences, and Causes of a Growing Public Health Problem. Curr. Obes. Rep..

[2] (2021). WHO. https://www.who.int/es/news-room/fact-sheets/detail/obesity-and-overweight.

[3] NCD Risk Factor Collaboration (NCD-RisC) (2017). Worldwide Trends in Body-Mass Index, Underweight, Overweight, and Obesity from 1975 to 2016: A Pooled Analysis of 2416 Population-Based Measurement Studies in 128.9 Million Children, Adolescents, and Adults. Lancet.

[4] Lindberg L., Danielsson P., Persson M., Marcus C., Hagman E. (2020). Association of childhood obesity with risk of early all-cause and cause-specific mortality: A Swedish prospective cohort study. PLoS Med..

[5] Fontaine K.R., Redden D.T., Wang C., Westfall A.O., Allison D.B. (2003). Years of Life Lost Due to Obesity. JAMA.

[6] Hagman E., Danielsson P., Elimam A., Marcus C. (2019). The effect of weight loss and weight gain on blood pressure in children and adolescents with obesity. Int. J. Obes..

[7] Tagi V.M., Chiarelli F. (2020). Obesity and insulin resistance in children. Curr. Opin. Pediatr..

[8] Valaiyapathi B., Gower B., Ashraf A.P. (2020). Pathophysiology of Type 2 Diabetes in Children and Adolescents. Curr. Diabetes Rev..

[9] Mann J.P., Valenti L., Scorletti E., Byrne C.D., Nobili V. (2018). Nonalcoholic Fatty Liver Disease in Children. Semin. Liver Dis..

[10] DeBoer M.D. (2019). Assessing and Managing the Metabolic Syndrome in Children and Adolescents. Nutrients.

[11] Reinert K.R.S., Po’E E.K., Barkin S.L. (2013). The Relationship between Executive Function and Obesity in Children and Adolescents: A Systematic Literature Review. J. Obes..

[12] Ronan L., Alexander-Bloch A., Fletcher P.C. (2019). Childhood Obesity, Cortical Structure, and Executive Function in Healthy Children. Cereb. Cortex.

[13] Shields C.V., Hultstrand K.V., West C.E., Gunstad J.J., Sato A.F. (2022). Disinhibited Eating and Executive Functioning in Children and Adolescents: A Systematic Review and Meta-Analysis. Int. J. Environ. Res. Public Health.

[14] Likhitweerawong N., Khorana J., Boonchooduang N., Phinyo P., Patumanond J., Louthrenoo O. (2022). Association between executive function and excess weight in pre-school children. PLoS ONE.

[15] Anderson J.R. (2010). Cognitive Psychology and Its Implications.

[16] Diamond A. (2013). Executive functions. Annu. Rev. Psychol..

[17] Best J.R., Miller P.H., Jones L.L. (2009). Executive functions after age 5: Changes and correlates. Dev. Rev..

[18] Li N., Yolton K., Lanphear B.P., Chen A., Kalkwarf H.J., Braun J.M. (2018). Impact of Early-Life Weight Status on Cognitive Abilities in Children. Obesity.

[19] Tee J.Y.H., Gan W.Y., Tan K.-A., Chin Y.S. (2018). Obesity and unhealthy lifestyle associated with poor executive function among Malaysian adolescents. PLoS ONE.

[20] Mamrot P., Hanć T. (2019). The association of the executive functions with overweight and obesity indicators in children and adolescents: A literature review. Neurosci. Biobehav. Rev..

[21] Favieri F., Forte G., Casagrande M. (2019). The Executive Functions in Overweight and Obesity: A Systematic Review of Neuro-psychological Cross-Sectional and Longitudinal Studies. Front. Psychol..

[22] García-Cáceres C., Quarta C., Varela L., Gao Y., Gruber T., Legutko B., Jastroch M., Johansson P., Ninkovic J., Yi C.-X. (2016). Astrocytic Insulin Signaling Couples Brain Glucose Uptake with Nutrient Availability. Cell.

[23] Dantzer R., O’Connor J.C., Freund G.G., Johnson R.W., Kelley K.W. (2008). From inflammation to sickness and depression: When the immune system subjugates the brain. Nat. Rev. Neurosci..

[24] King K.P., Keller C.V., Evans C.T., Murdaugh D.L., Gower B.A., Gowey M.A. (2022). Inflammation, Executive Function, and Adiposity in Children With or at Risk for Obesity: A Pilot Study. J. Pediatr. Psychol..

[25] Arnoldussen I.A., Kiliaan A.J., Gustafson D.R. (2014). Obesity and dementia: Adipokines interact with the brain. Eur. Neuropsychopharmacol..

[26] McNay E.C., Ong C.T., McCrimmon R.J., Cresswell J., Bogan J.S., Sherwin R.S. (2010). Hippocampal memory processes are modulated by insulin and high-fat-induced insulin resistance. Neurobiol. Learn. Mem..

[27] Bruce-Keller A.J., Salbaum J.M., Berthoud H.-R. (2017). Harnessing Gut Microbes for Mental Health: Getting From Here to There. Biol. Psychiatry.

[28] Davis C.L., Tomporowski P.D., McDowell J.E., Austin B.P., Miller P.H., Yanasak N.E., Allison J.D., Naglieri J.A. (2011). Exercise improves executive function and achievement and alters brain activation in overweight children: A randomized, controlled trial. Health Psychol..

[29] Ortega F.B., Mora-Gonzalez J., Cadenas-Sanchez C., Esteban-Cornejo I., Migueles J.H., Solis-Urra P., Verdejo-Román J., Rodriguez-Ayllon M., Molina-Garcia P., Ruiz J.R. (2022). Effects of an Exercise Program on Brain Health Outcomes for Children With Overweight or Obesity. JAMA Netw. Open.

[30] Crova C., Struzzolino I., Marchetti R., Masci I., Vannozzi G., Forte R., Pesce C. (2013). Cognitively challenging physical activity benefits executive function in overweight children. J. Sports Sci..

[31] Krafft C.E., Schaeffer D.J., Schwarz N.F., Chi L., Weinberger A.L., Pierce J.E., Rodrigue A.L., Allison J.D., Yanasak N.E., Liu T. (2014). Improved Frontoparietal White Matter Integrity in Overweight Children Is Associated with Attendance at an After-School Exercise Program. Dev. Neurosci..

[32] Kleinloog J.P.D., Mensink R.P., Ivanov D., Adam J.J., Uludağ K., Joris P.J. (2019). Aerobic Exercise Training Improves Cerebral Blood Flow and Executive Function: A Randomized, Controlled Cross-Over Trial in Sedentary Older Men. Front. Aging Neurosci..

[33] Knight S.P., Laird E., Williamson W., O’connor J., Newman L., Carey D., De Looze C., Fagan A.J., Chappell M.A., Meaney J.F. (2021). Obesity is associated with reduced cerebral blood flow—Modified by physical activity. Neurobiol. Aging.

[34] Colcombe S.J., Erickson K.I., Scalf P.E., Kim J.S., Prakash R., McAuley E., Kramer A.F. (2006). Aerobic exercise training increases brain volume in aging humans. J. Gerontol. Ser. A Biol. Sci. Med. Sci..

[35] Dishman R.K., Berthoud H., Booth F.W., Cotman C.W., Edgerton V.R., Fleshner M.R., Gandevia S.C., Gomez-Pinilla F., Greenwood B.N., Hillman C.H. (2006). Neurobiology of Exercise. Obesity.

[36] Cotman C.W., Berchtold N.C. (2002). Exercise: A behavioral intervention to enhance brain health and plasticity. Trends Neurosci..

[37] Bourbeau K., Zuhl M., Gibson A., Kravitz L., Mermier C. (2023). Mechanisms of aerobic exercise in attenuating obesity-induced cognitive impairment: A brief review. Obes. Med..

[38] Bustamante E.E., Williams C.F., Davis C.L. (2016). Physical Activity Interventions for Neurocognitive and Academic Performance in Overweight and Obese Youth. Pediatr. Clin. N. Am..

[39] Sun X., Li Y., Cai L., Wang Y. (2020). Effects of physical activity interventions on cognitive performance of overweight or obese children and adolescents: A systematic review and meta-analysis. Pediatr. Res..

[40] Martin A., Booth J.N., Laird Y., Sproule J., Reilly J.J., Saunders D.H. (2018). Physical activity, diet and other behavioural interventions for improving cognition and school achievement in children and adolescents with obesity or overweight. Cochrane Database Syst. Rev..

[41] Bramer W.M., Rethlefsen M.L., Kleijnen J., Franco O.H. (2017). Optimal database combinations for literature searches in systematic reviews: A prospective exploratory study. Syst. Rev..

[42] Zhao J.-G. (2014). Combination of multiple databases is necessary for a valid systematic review. Int. Orthop..

[43] Moher D., Shamseer L., Clarke M., Ghersi D., Liberati A., Petticrew M., Shekelle P., Stewart L.A., PRISMA-P Group (2015). Preferred reporting items for systematic review and meta-analysis protocols (prisma-p) 2015 statement. Syst. Rev..

[44] Xie C., Wang X., Zhou C., Xu C., Chang Y.-K. (2017). Exercise and dietary program-induced weight reduction is associated with cognitive function among obese adolescents: A longitudinal study. PeerJ.

[45] Page M.J., McKenzie J.E., Bossuyt P.M., Boutron I., Hoffmann T.C., Mulrow C.D., Shamseer L., Tetzlaff J.M., Akl E.A., Brennan S.E. (2021). The PRISMA 2020 statement: An updated guideline for reporting systematic reviews Systematic reviews and Meta-Analyses. BMJ.

[46] Suzuki H., Sakuma N., Kobayashi M., Ogawa S., Inagaki H., Edahiro A., Ura C., Sugiyama M., Miyamae F., Watanabe Y. (2022). Normative Data of the Trail Making Test Among Urban Community-Dwelling Older Adults in Japan. Front. Aging Neurosci..

[47] Frost A., Moussaoui S., Kaur J., Aziz S., Fukuda K., Niemeier M. (2021). Is the n-back task a measure of unstructured working memory capacity? Towards understanding its connection to other working memory tasks. Acta Psychol..

[48] Barzykowski K., Wereszczyński M., Hajdas S., Radel R. (2022). Cognitive inhibition behavioral tasks in online and laboratory settings: Data from Stroop, SART and Eriksen Flanker tasks. Data Brief.

[49] Contreras-Osorio F., Ramirez-Campillo R., Cerda-Vega E., Campos-Jara R., Martínez-Salazar C., Araneda R., Ebner-Karestinos D., Arellano-Roco C., Campos-Jara C. (2022). Effects of Sport-Based Exercise Interventions on Executive Function in Older Adults: A Systematic Review and Meta-Analysis. Int. J. Environ. Res. Public Health.

[50] Contreras-Osorio F., Ramirez-Campillo R., Cerda-Vega E., Campos-Jara R., Martínez-Salazar C., Reigal R.E., Morales-Sanchez V., Sierralta S.A., Campos-Jara C. (2022). Effects of Physical Exercise on Executive Function in Adults with Depression: A Systematic Review and Meta-Analysis Protocol. Sustainability.

[51] Drevon D., Fursa S.R., Malcolm A.L. (2016). Intercoder Reliability and Validity of WebPlotDigitizer in Extracting Graphed Data. Behav. Modif..

[52] Lane B., McCullagh R., Cardoso J.R., McVeigh J.G. (2022). The effectiveness of group and home-based exercise on psychological status in people with ankylosing spondylitis: A systematic review and meta-analysis. Musculoskelet. Care.

[53] Xia H.-S., Li Y.-X., Zhang Q.-Y., Zhong D.-L., Liu X.-B., Gou X.-Y., Fan J., Zhao J., Zhang Y., Ai S.-C. (2023). Attention bias modification for depression: A systematic review and meta-analysis. Front. Psychiatry.

[54] Zadro S., Stapleton P. (2022). Does Reiki Benefit Mental Health Symptoms Above Placebo?. Front. Psychol..

[55] Sterne J.A.C., Sutton A.J., Ioannidis J.P.A., Terrin N., Jones D.R., Lau J., Carpenter J., Rücker G., Harbord R.M., Schmid C.H. (2011). Recommendations for examining and interpreting funnel plot asymmetry in meta-analyses of randomised controlled trials. BMJ.

[56] Balshem H., Helfand M., Schünemann H.J., Oxman A.D., Kunz R., Brozek J., Vist G.E., Falck-Ytter Y., Meerpohl J., Norris S. (2011). GRADE guidelines: 3. Rating the quality of evidence. J. Clin. Epidemiol..

[57] Guyatt G., Oxman A.D., Akl E.A., Kunz R., Vist G., Brozek J., Norris S., Falck-Ytter Y., Glasziou P., DeBeer H. (2011). GRADE guidelines: 1. Introduction—GRADE evidence profiles and summary of findings tables. J. Clin. Epidemiol..

[58] García-Hermoso A., Ramírez-Campillo R., Izquierdo M. (2019). Is Muscular Fitness Associated with Future Health Benefits in Children and Adolescents? A Systematic Review and Meta-Analysis of Longitudinal Studies. Sports Med..

[59] Moran J., Ramirez-Campillo R., Granacher U. (2018). Effects of Jumping Exercise on Muscular Power in Older Adults: A Meta-Analysis. Sports Med..

[60] Abt G., Boreham C., Davison G., Jackson R., Wallace E., Williams A.M. (2021). Registered Reports in the Journal of Sports Sciences. J. Sports Sci..

[61] Hopkins W., Marshall S., Batterham A., Hanin J. (2009). Progressive statistics for studies in sports medicine and exercise science. Med. Sci. Sports Exerc..

[62] Kadlec D., Sainani K.L., Nimphius S. (2022). With Great Power Comes Great Responsibility: Common Errors in Meta-Analyses and Meta-Regressions in Strength & Conditioning Research. Sports Med..

[63] Higgins J.P.T., Thompson S.G. (2002). Quantifying heterogeneity in a meta-analysis. Stat. Med..

[64] Egger M., Smith G.D., Schneider M., Minder C. (1997). Bias in meta-analysis detected by a simple, graphical test. BMJ.

[65] Duval S., Tweedie R. (2000). Trim and Fill: A Simple Funnel-Plot-Based Method of Testing and Adjusting for Publication Bias in Meta-Analysis. Biometrics.

[66] Shi L., Lin L. (2019). The trim-and-fill method for publication bias: Practical guidelines and recommendations based on a large database of meta-analyses. Medicine.

[67] Song Y., Fan B., Wang C., Yu H. (2023). Meta-analysis of the effects of physical activity on executive function in children and adolescents with attention deficit hyperactivity disorder. PLoS ONE.

[68] Liu S., Yu Q., Li Z., Cunha P.M., Zhang Y., Kong Z., Lin W., Chen S., Cai Y. (2020). Effects of Acute and Chronic Exercises on Executive Function in Children and Adolescents: A Systemic Review and Meta-Analysis. Front. Psychol..

[69] Singh A.S., Saliasi E., Van Den Berg V., Uijtdewilligen L., De Groot R.H.M., Jolles J., Andersen L.B., Bailey R., Chang Y.-K., Diamond A. (2019). Effects of physical activity interventions on cognitive and academic performance in children and adolescents: A novel combination of a systematic review and recommendations from an expert panel. Br. J. Sports Med..

[70] Wassenaar T.M., Williamson W., Johansen-Berg H., Dawes H., Roberts N., Foster C., Sexton C.E. (2020). A critical evaluation of systematic reviews assessing the effect of chronic physical activity on academic achievement, cognition and the brain in children and adolescents: A systematic review. Int. J. Behav. Nutr. Phys. Act..

[71] Jebeile H., Kelly A.S., O’Malley G., Baur L.A. (2022). Obesity in children and adolescents: Epidemiology, causes, assessment, and management. Lancet Diabetes Endocrinol..

