# Supplementary material for: Physical Exercise and Executive Function in the Pediatric Overweight and Obesity Population: A Systematic Review Protocol
Source: Sports (Basel). 2024 Jun 26;12(7):180. doi: 10.3390/sports12070180 (PMC11280794; doi:10.3390/sports12070180)
Supplement: Supplementary file 1 [file sports-12-00180-s001.zip › Table S2.pdf]

Table S2

Terms to be used in the search strategy according to each database.

Database	Search strategy
EBSCO	AB "Executive Function" OR AB "Cognitive Function" OR AB Cognition OR AB "Inhibitory control" OR AB Inhibition OR AB "Working memory" OR AB "Executive functioning" OR AB "Cognitive flexibility" AND AB Sport OR AB "Modified sport" OR AB Fitness OR AB Exercise OR AB "Physical Activity" OR AB Athletics OR AB "Sport Practice" AND AB obesity OR AB overweight
PubMed	(((((((((("Cognitive flexibility"[Title/Abstract]) OR ("Executive functioning"[Title/Abstract])) OR ("Working memory"[Title/Abstract])) OR (Inhibition[Title/Abstract])) OR ("Inhibitory control"[Title/Abstract])) OR (Cognition[Title/Abstract])) OR ("Cognitive Function"[Title/Abstract])) OR ("Executive Function"[Title/Abstract]))) AND ((((((("Sport Practice"[Title/Abstract]) OR (Athletics[Title/Abstract])) OR ("Physical Activity"[Title/Abstract])) OR (Exercise[Title/Abstract])) OR (Fitness[Title/Abstract])) OR ("Modified sport"[Title/Abstract])) OR (Sport[Title/Abstract]))) AND ((overweight[Title/Abstract]) OR (obesity[Title/Abstract]))
Scopus	(TITLE-ABS-KEY ("executive function") OR TITLE-ABS-KEY ("cognitive function") OR TITLE-ABS-KEY (cognition) OR TITLE-ABS-KEY ("inhibitory control") OR TITLE-ABS-KEY ("working memory") OR TITLE-ABS-KEY ("executive functioning") OR TITLE-ABS-KEY ("cognitive flexibility") AND TITLE-ABS-KEY ("modified sport") OR TITLE-ABS-KEY (fitness) OR TITLE-ABS-KEY (exercise) OR TITLE-ABS-KEY ("physical activity") OR TITLE-ABS-KEY (athletics) OR TITLE-ABS-KEY ("sport practice") AND TITLE-ABS-KEY (obesity) OR TITLE-ABS-KEY (overweight))
Web of Science	1: AB=("executive function")<br>2: AB=("cognitive function")<br>3: AB=(cognition)<br>4: AB=("inhibitory control")<br>5: AB=("working memory")<br>6: AB=("executive functioning")<br>7: AB=("cognitive flexibility")<br>8: #1 OR #2 OR #3 OR #4 OR #5 OR #6 OR #7<br>9: AB=(sport)<br>10: AB=("modified sport")<br>11: AB=(fitness)<br>12: AB=(exercise)<br>13: AB=("physical activity")<br>14: AB=(athletics)

	15: AB=("sport practice")<br>16: #9 OR #10 OR #11 OR #12 OR #13 OR #14 OR #15<br>17: AB=(obesity)<br>18: AB=(overweight)<br>19: #17 OR #18<br>20: #8 AND #16 AND #19
